# Influenza AH1N2 Viruses, United Kingdom, 2001–02 Influenza Season

**DOI:** 10.3201/eid0903.020404

**Published:** 2003-03

**Authors:** Joanna S. Ellis, Adriana Alvarez-Aguero, Vicky Gregory, Yi Pu Lin, A. Hay, Maria C. Zambon

**Affiliations:** * Central Public Health Laboratory, London, United Kingdom; † National Institute for Medical Research, London, United Kingdom

**Keywords:** influenza, H1N2, reassortment, evolution, surveillance, diagnosis, research

## Abstract

During the winter of 2001–02, influenza AH1N2 viruses were detected for the first time in humans in the U.K. The H1N2 viruses co-circulated with H3N2 viruses and a very small number of H1N1 viruses and were isolated in the community and hospitalized patients, predominantly from children <15 years of age. Characterization of H1N2 viruses indicated that they were antigenically and genetically homogeneous, deriving the hemagglutinin (HA) gene from recently circulating A/New Caledonia/20/99-like H1N1 viruses, whereas the other seven genes originated from recently circulating H3N2 viruses. Retrospective reverse transcription-polymerase chain reaction analysis of influenza A H1 viruses isolated in the U.K. during the previous winter identified a single H1N2 virus, isolated in March 2001, indicating that H1N2 viruses did not widely circulate in the U.K. before September 2001. The reassortment event is estimated to have occurred between 1999 and early 2001, and the emergence of H1N2 viruses in humans reinforces the need for frequent surveillance of circulating viruses.

The influenza A virus genome consists of eight single-stranded RNA segments of negative sense. The segmented nature of the genome allows reassortment of genes between different influenza A strains infecting one host, which may generate novel antigenic variants and give rise to pandemics of disease in humans. Although the influenza pandemic of 1918 appears to have followed the introduction of an avian-like H1N1 virus into the human population (*1*), the H2N2 and H3N2 viruses responsible for the 1957 and 1968 human pandemics, respectively, were generated by reassortment between human and avian viruses (*2*–*4*). Since the last influenza pandemic of 1977, which was caused by the reemergence of the H1N1 subtype, two subtypes of influenza A (H1N1 and H3N2) have been co-circulating in humans together with influenza B viruses.

The co-circulation of influenza A H1N1 and H3N2 viruses in humans has led to sporadic reports of the isolation of H1N1-H3N2 reassortant viruses in humans (*5*–*10*). In contrast, after their isolation from pigs in 1994 (*11*,*12*), influenza A H1N2 reassortant viruses, derived from human and avian viruses, have become established in swine throughout the U.K. Influenza viruses of H1N2 subtype, derived from genetic reassortment of strains endemic in pigs, have also been established in pigs in Japan since 1978 (*13*,*14*) and, more recently, in France (*15*) and North America (*16*).

During 1988–89, several H1N2 viruses were isolated from humans in China (*9*,*10*). Genetic analysis of these reassortant viruses indicated that only the hemagglutinin (HA) gene was derived from the prevailing human H1N1 virus, whereas all other genes, including the neuraminidase (NA) gene, were introduced from the prevailing human H3N2 strain. These reassortant viruses did not spread to other countries until 2001; further isolations of influenza A H1N2 viruses in humans have not been documented.

Influenza AH1N2 viruses were detected for the first time in humans in the U.K. during the winter of 2001–02. We examine the diversity of H1N2 viruses emerging in the U.K. over a 12-month period in 2001–02. The antigenic and genetic properties of these viruses are described, as well as the relationship of reassortant viruses to parental H1N1 and H3N2 strains. Furthermore, the impact of the circulation of H1N2 viruses on disease in the community is considered, as well as the implications for diagnostic testing of influenza strains.

## Materials and Methods

### Epidemiologic Data

Data on the rates of community cases of influenza and influenzalike illness were collected by the Birmingham Unit of the Royal College of General Practitioners and are based on weekly returns from approximately 69 sentinel practices throughout England and Wales. The Communicable Disease Surveillance Centres in Wales and Northern Ireland and the Scottish Centre for Infection and Environmental Health provided additional data. These three mechanisms for monitoring clinical incidence of influenzalike illness cover a total population of 800,000 persons. Incidence data were recorded as new physician consultations per 100,000 persons.

### Source of Isolates

Influenza viruses isolated in hospital laboratories throughout the U.K. were sent to the Enteric, Respiratory and Neurological Laboratory at the Central Public Health Laboratory for antigenic characterization. Throughout the influenza season (October–March), a clinical practices group in the Birmingham Unit of the Royal College of General Practitioners network obtained nose and throat swabs from patients who had influenzalike illness (*17*), which were sent directly by mail to Enteric, Respiratory and Neurological Laboratory for virus isolation and subsequent antigenic and genetic characterization. All specimens were given a laboratory sample number upon arrival.

### Clinical Sample Injection

Combined nose and throat swab specimens in virus transport medium were treated as previously described and injected onto confluent Madin-Darby canine kidney cells (*18*). The cells were incubated at 33°C, and the medium was tested at day 7 for HA of turkey red cells.

### Virus Typing

Influenza viruses were typed by using ferret antisera in hemagglutination inhibition tests as described (*19*). All HI tests were carried out by using 8 HA U of virus and 0.5% (v/v) turkey red blood cells. All ferret sera were treated with receptor-destroying enzyme. After typing, virus isolates were given a unique strain designation number in strict chronologic order.

### Amantadine Sensitivity Assays

Susceptibility of influenza virus replication to inhibition by amantadine was determined by virus infectivity assays (*20*). Madin-Darby canine kidney cells were overlaid with medium containing amantadine (SigmaAldrich, Poole, Dorset, England) at a concentration of 0.1, 1.0, or 10.0 µg/mL.

### Reverse Transcription-Polymerase Chain Reaction (RT-PCR) and Nucleotide Sequencing

Viral RNA was extracted from 15-µL samples by using the MagNA Pure LC total nucleic acid isolation kit, on a MagNA Pure LC extraction robot (Roche Molecular Biochemicals, Mannheim, Germany) (*21*). Reverse transcription was performed as previously described (*20*). PCR to detect N1 and N2 neuraminidase genes was performed with nested primer sets (primer sequences available on request), modified from previously described assays (*22*).

Viruses selected for genetic analysis are shown in Table 1. Amplification of the HA1 domain of the HA gene, the complete coding region of the NA gene and portions of the PB2, PB1, PA, NP, NS, and M genes required primers specific for the eight genes (primer sequences available by request). The regions amplified for sequence analysis were PB2, 46-487; PB1, 970-1427; PA, 499-817; HA, 84-1058; NP, 1045-1428; NA, 20-1426; M, 249-612; and NS, 146-843. PCR products were purified by using agarose gel electrophoresis and a QIAquick gel extraction kit (Qiagen Ltd, Crawley, West Sussex, England) and sequenced by using a Beckman Coulter CEQ 2000 capillary sequencer and CEQ 2000 Dye Terminator cycle sequencing Quick Start kit (Beckman Coulter, Fullerton, CA). The fragment lengths compared were PB2, 442; PB1, 458; PA, 319; HA, 975; NP, 384; NA, 1407; M, 364; and NS, 698 nucleotides. Nucleotide sequences determined in this study are available from the European Molecular Biology Laboratory database (accession nos. AJ489485–AJ489502, AJ489530–AJ489559, and AJ489846–AJ489862). The nucleotide sequences for the NA, M1, and NP of A/Panama/2007/99 and A/Moscow/10/99 viruses are available in the European Molecular Biology Laboratory database under accession numbers AJ457937, AJ457966, AJ458298, AJ458297, AJ458268, and AJ458267 (*23*).

**Table 1:** Influenza A H1N1 and H1N2 U.K. viruses selected for genetic analysis

Virus	Sample date	Region	Source	Gene analyzed
**H1N1**				
A/England161/2002	9/2/02	South	Hospital	HA
**H1N2**				
A/England/627/2001	19/3/01	South	Hospital	PB2, PB1, HA, NP, NA, M, NS
A/Scotland/122/2001	27/9/01	Scotland	Hospital	PB2, PB1, HA, NP, M
A/England/689/2001	24/12/01	Not known	Hospital	HA
A/England/691/2001	21/12/01	Central	Hospital	PB2, PB1, PA, HA, NP, NA, M, NS
A/England/1/2002	4/1/02	Central	Hospital	NA
A/England/3/2002	8/1/02	Central	Hospital	PB2, PB1, NP, M, NS
A/England/5/2002	9/1/02	Central	Community	PB2, PB1, PA, NP, M, NS
A/England18/2002	15/1/02	Central	Community	HA
A/England/57/2002	16/1/02	South	School	PB2, PB1, PA, NP, NA, M, NS
A/England/73/2002	30/1/02	South	School	PB2, PA, NP, M, NS
A/England/90/2002	24/1/02	North	School	PB2, PB1, PA, NP, M, NS
A/England/97/2002	1/2/02	North	Hospital	PB2, PB1, NP, NA, M, NS
A/Scotland/15/2002	19/2/02	Scotland	Hospital	HA
575/2001^a^	13/12/01	Central	Hospital	HA
576/2001^a^	13/12/01	Central	Hospital	HA
1352/2002^a^	24/1/02	North	School	HA
1496/2002^a^	30/1/02	North	Community	NA
1660/2002^a^	6/2/02	Central	Hospital	HA

^a^Indicates where virus was not isolated by culture from polymerase chain reaction-positive material.

### Phylogenetic Analysis

Sequences were analyzed by neighbor joining with the Phylip (version 3.57) suite of programs (DNADist and Fitch) and bootstrapping by Seqboot (*24*). Bootstrap values >70 were regarded as statistically significant.

## Results

### Epidemiology

**Figure 1:**
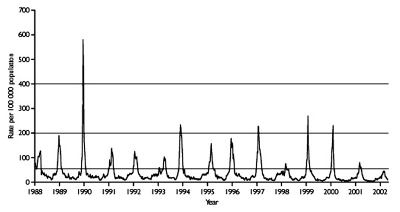
Consultation rate (per 100,000 population) for influenzalike illnesses with sentinel physicians in England in 1988–2002 (from the Royal College of General Practitioners Weekly Returns Service). Baseline activity is defined by a consultation rate <50/100,000; normal seasonal activity, 50–200/100,000; higher than seasonal activity, 200–400/100,000, and epidemic activity is defined as >400/100,000 population.

During the period September 2001–March 2002, clinical death indices for the measurement of influenzalike illness in England and Wales did not rise above baseline levels, indicating low influenza activity (Figure 1). Very low levels of influenza activity were also recorded in Scotland (data not shown). The clinical indices of influenzalike illness activity peaked in week 6 and correlated well with the peak of influenza viruses detected and typed by Central Public Health Laboratory (data not shown; available from: URL: http://www.phls.co.uk).

In September 2001, the first isolate of the influenza season (A/Scotland/122/2001), an H1N2 virus, was isolated from a 1-year-old child. H1N2 viruses continued to be isolated from the patients in the community and in hospitalized patients throughout the U.K. until the end of March 2002. H1N2 reassortant viruses co-circulated with a relatively equal proportion of H3N2 viruses throughout the season and a small number of H1N1 viruses. Of 420 influenza A viruses isolated and characterized in the winter of 2001–02, 54.0% were influenza A H1N2, 45.2% H3N2, and 0.7% A H1N1 subtype.

During the previous influenza season (2000–01), H1N1 and influenza B viruses co-circulated in the U.K., and no H3N2 viruses were detected. In comparison, only a small number of influenza B viruses were isolated during the winter of 2001–02 (data not shown; available from: URL: http://www.phls.co.uk).

Retrospective analysis using RT-PCR to determine the NA subtype, on 198 of 323 influenza A H1 viruses isolated during 2000–01, identified only a single H1N2 subtype virus isolated from a 9-year-old child in March 2001. Therefore, before September 2001, H1N2 viruses were not widely circulating in the U.K.

### Clinical Impact of H1N2 Viruses

**Figure 2:**
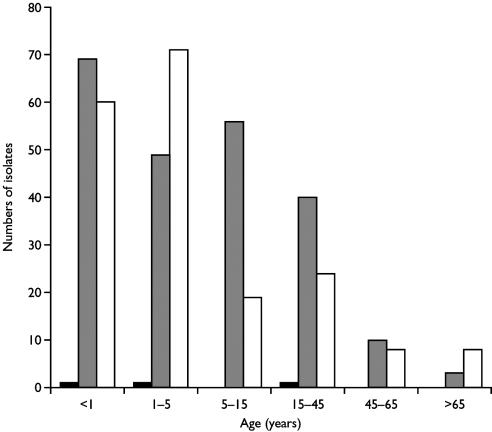
Age distribution of patients from whom influenza A viruses isolated during 2001–02 in the U.K. ■H1N1 isolates, ■H1N2 isolates, □H3N2 isolates.

Despite the fact that H1N2 viruses emerged and were as frequently isolated as H3N2 viruses during the winter of 2001–02 in England, the levels of clinical influenzalike illness activity were among the lowest in the last 15 years (Figure 1). This low level indicates that the new H1N2 strain was not associated with particularly severe influenzalike illness activity. The low levels of influenzalike illness activity were also associated with correspondingly low levels of excess death rates (death from all causes), (available from: URL: http://www.phls.co.uk). More than 75% of H1N2 virus isolates were obtained from children <15 years of age (Figure 2), indicating that the major age group affected by the H1N2 viruses was young persons, possibly undergoing a primary infection. A similar proportion of H3N2 viruses isolated were also from children <15 years of age. Few H1N2 virus isolates were obtained from adults, and only a limited number were obtained from adults >65 years old. These facts suggest that the young are most susceptible to H1N2; adults and vaccinated elderly appear to have adequate protective immunity to the new subtype. Age-specific consultation data for influenzalike illness in 2001–02 confirm that the age range most severely affected by influenzalike illness was the 5–14 age group (data not shown; available from: URL: http://www.phls.co.uk).

### Antigenic Analysis of H1N2 Viruses

Antigenic characterization of the 228 H1N2 viruses isolated during 2001–02 was performed by using hemagglutination inhibition tests with postinfection ferret antisera to influenza A H1N1 and H3N2 reference and vaccine strains (Table 2). The HA gene of the H1N2 viruses was antigenically related to that of the H1N1 strain used in vaccines in 2001–02, A/NewCaledonia/20/99, and was antigenically indistinguishable from that of co-circulating A/NewCaledonia/20/99-like H1N1 viruses. No substantial indication of antigenic drift in the HA genes of these viruses was observed over the period the viruses were isolated (March 2001–March 2002) (Table 2).

**Table 2:** Antigenic analysis of influenza A H1N2 by hemagglutination inhibition assays

		Post-infection ferret antisera				
Virus	Sample date	A/Wuhan 371/95	A/Bay 7/95	A/NewCal 20/99	A/Moscow 10/99	A/Pan 2007/99
**H1N1**						
A/Wuhan/371/95		1,280	320	80	<40	<40
A/Bayern7/95		80	5,120	1,280	<40	<40
A/NewCaled/20/99		160	80	5,120	<40	<40
A/England/70/2002	29/1/02	160	40	1,280	<40	<40
A/England/47/2002	29/1/02	80	40	320	<40	<40
**H1N2**						
A/England/627/2001	19/3/01	160	80	640	<40	<40
A/Scotland/122/2001	27/9/01	160	160	2,560	<40	<40
A/England/691/2001	18/12/01	80	40	1,280	<40	<40
A/England/46/2002	29/1/02	40	<40	320	<40	<40
A/England/334/2002	6/3/02	80	<40	1,280	<40	<40
**H3N2**						
A/Moscow/10/99		<40	<40	<40	640	320
A/Panama/2007/99		<40	<40	<40	80	640
A/England/687/2001	29/11/01	<40	<40	<40	2,560	1,280
A/England/12/2002	16/1/02	<40	<40	<40	2,560	320
A/Scotland/2/2002	23/1/02	<40	<40	<40	5,120	1,280

### Sequence Analysis of HA and NA Genes

**Figure 3:**
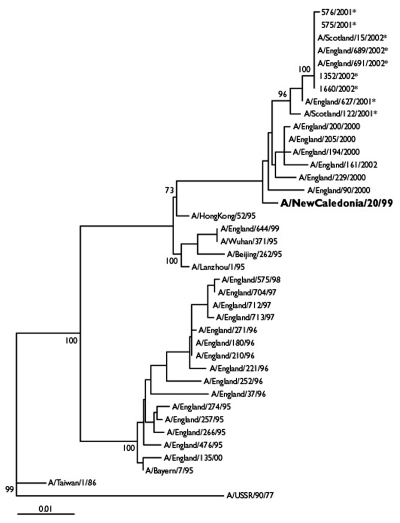
Phylogenetic tree of influenza A H1N1 and H1N2 virus HA1 nucleotide sequences. The tree was generated by using joining-joining analysis. The lengths of the horizontal lines are proportional to the number of nucleotide substitutions per site. Trees were bootstrapped x100. H1N2 viruses are indicated with an asterisk. The current H1N1 vaccine strain is in bold typeface.

Phylogenetic analysis indicated that the HA1 sequences of the H1N2 viruses analyzed (Table 1) were most closely related (sequence similarity of 98.7% to 99.1%) to those of the H1N1 vaccine strain, A/NewCaledonia/20/99 (Figure 3). The HA1 sequences of the H1N2 viruses analyzed were very similar, exhibiting only 0%–1.2% nucleotide divergence between sequences. This divergence is in contrast to that of A/NewCaledonia/20/99-like H1N1 viruses isolated in the U.K. during 2000–01, which exhibited 0.3%–3.1% nucleotide divergence in the HA1.

Of the four amino acid differences previously observed in the HA1 sequences of H1N2 viruses isolated from various countries and compared to A/NewCaledonia/20/99 (*23*), all U.K. H1N2 viruses analyzed had the substitutions V169A and A193T. All but the earliest U.K. H1N2 viruses had the substitutions V178I and A218T in the HA1.

The NA genes of six H1N2 viruses analyzed (Table 1) were closely related genetically to the NA genes of recently circulating H3N2 viruses, represented by A/Moscow/10/99 (99.1% nucleotide sequence homology). All analyzed H1N2 NA gene sequences from the U.K. differed from those of A/Moscow/10/99 at the three amino acid positions observed in the NA of H1N2 viruses isolated from other parts of the world (*23*). In addition, with the exception of the earliest U.K. H1N2 isolate (A/England/627/2001), all analyzed H1N2 NA sequences from the U.K. had the substitution M24T, which is located in the transmembrane region of the protein (*25*).

We observed no mutations in the NA or HA genes that have been reported to confer resistance to NA inhibitors (*26*,*27*). None of the amino acid substitutions in the HA or NA genes resulted in the loss or creation of potential glycosylation sites in the HA or NA surface proteins of the H1N2 viruses.

### Genetic Variation in the Internal Protein Genes

A region of the M1 gene of nine H1N2 isolates (Table 1) was analyzed and compared with sequences of prototype H1N1, H2N2, and H3N2 viruses, in addition to M1 gene sequence data obtained from influenza viruses isolated in the U.K. since 1951. Little sequence divergence was observed between the M1 sequences of the H1N2 viruses (0% to 0.9%), and they were most closely related to those of recent H3N2 viruses. No amino acid differences were seen between the partial M1 sequences of H1N2 viruses and those of A/Panama/2007/99, whereas one amino acid difference (R174K) was found from the M1 of A/Moscow/10/99. This substitution was first observed in H3N2 strains isolated in England in 1996 and was fixed in H3N2 viruses isolated after 1998 in the U.K.

Partial gene sequencing of the PB2, PB1, PA, NP, and NS genes indicated that the analyzed H1N2 viruses (Table 1) also derived each of these genes from a virus of the H3N2 subtype. Very little sequence divergence was observed in these five genes of the H1N2 viruses analyzed.

### Amantadine Sensitivity

The susceptibility of H1N2 virus replication to inhibition by amantadine was determined. All of the analyzed H1N2 viruses (A/Scotland/122/2001, A/England/45/2002, and A/England/63/2002) were susceptible to inhibition of virus growth by amantadine, with MIC 50 <0.1µg/mL of drug.

## Discussion

In the winter of 2001–02, which was a mild influenza season, influenza A H1N2 viruses were detected for the first time in the U.K. Analysis of the diversity of >200 H1N2 isolates from the U.K indicated that they were closely related antigenically and genetically and derived the HA gene from A/NewCaledonia/20/99-like H1N1 viruses, whereas the other seven genes originated from recently circulating H3N2 viruses. The H1N2 viruses were isolated throughout the influenza season and co-circulated with H3N2 viruses. In contrast to the previous winter’s results, few influenza A H1N1 viruses were detected. Retrospective RT-PCR analysis of 61.4% of influenza AH1 viruses isolated during 2000–01 in the U.K. identified one H1N2 virus that had been isolated in March 2001, indicating that H1N2 viruses had not circulated widely in the U.K. before becoming established in autumn of 2001. H1N2 viruses have also been identified during 2001–02 from outbreaks of influenza in different countries in Africa, America, Asia, and Europe (*28*). The earliest H1N2 viruses identified worldwide retrospectively were also isolated in March 2001 in Saudi Arabia (*23*).

During the past decade, influenza A H1N1 viruses have circulated intermittently in the U.K (*29*). Between October 2000 and April 2001, a season of low influenza activity in the U.K., influenza A H1N1 viruses were the predominate influenza A strain circulating. Most H1N1 viruses were antigenically closely related to the vaccine strain used in the 2000–01 influenza season, A/NewCaledonia/20/99. The circulation of H1N2 reassortant viruses appears to have displaced A/NewCaledonia/20/99-like H1N1 viruses, although circulation of H1N2 does not appear to be associated with the generation of viruses with antigenically different surface proteins since the HA and NA of H1N2 viruses are antigenically similar to those of recently circulating H1N1 and H3N2 viruses. The observation that H1N2 viruses were isolated mainly from children <15 years of age suggests a preexisting immunity to H1 and N2 in the population (Figure 2). Furthermore, a similar proportion of the H3N2 viruses isolated during 2001–02 (79%) were also isolated from children <15 years old, with A/Panama/2007/99-like H3N2 viruses circulating during the same period of time as the A/NewCaledonia/20/99-like H1N1 viruses in the U.K. This conclusion is supported by the observational disease data in which the age group most severely affected by the influenza viruses circulating was the 5–14 age group and suggests that disease associated with both H1N2 and H3N2 was mainly in children acquiring a primary infection. In contrast, during the 2000–01 winter season, when influenza A H1N1 and influenza B viruses co-circulated, the disease data demonstrated that the age groups most affected by influenza viruses were the 5–14 years of age group and 15–44 years of age group (available from: URL: http://www.phls.co.uk). The age distribution of the disease data correlates with the finding that most H1N1 viruses isolated during 2000–01 were from the 5–14 years of age and 15–44 years of age groups.

Our analysis of genetic variation in the HA gene showed that seven amino acid substitutions have accumulated in the HA1 gene of influenza A H1N1 viruses between the circulation of A/Beijing/252/95-like H1N1 viruses in the U.K. and the emergence of viruses similar to the new variant, A/NewCaledonia/20/99, in October 2000. Of these, four mutations (T136S, E156G, S186P, and I194L) are located in two of the five antigenic sites (A and B) of HA1. The emergence of A/NewCaledonia/20/99–like viruses correlates with the finding that new drift variants of epidemiologic importance typically have four or more amino acid substitutions located in two or more of the antigenic sites (*30*). Additionally, the substitutions A193T and A218T are located in antigenic sites B and D, respectively, of HA1, indicating the potential for further drift in the HA gene of H1N2 viruses and the possible subsequent generation of a new epidemic variant.

Although H1N1 and H3N2 subtypes have co-circulated in the human population since 1977, reassortant combinations of HA and NA subtypes are rare. Reassortant H1N2 viruses have previously been isolated from sporadic cases in humans and were not maintained in circulation in humans (*9*). The H1 HA–N2 NA combination may provide a better functional match between receptor-binding and receptor-destroying activities of HA and NA, respectively, perhaps providing H1N2 viruses with a fitness advantage over contemporary H1N1, H3N2, or both viruses. Postreassortment mutations in the HA gene located in the vicinity of the receptor-binding pocket have previously been shown to compensate for any imbalance between HA and NA activities and may be a factor in influenza virus evolution (*31*,*32*). Analysis of the HA1 sequences of H1N1 and H1N2 viruses isolated between December 2000 and February 2002 in the U.K. showed three substitutions at positions 178, 193, and 218 that were present only in the HA gene of the H1N2 viruses analyzed; residue 193 situated near to the HA receptor binding pocket. As these three mutations were not observed in the HA1 of recent H1N1 viruses analyzed, the substitutions may have occurred in H1N2 viruses after reassortment.

The NA genes of the H1N2 viruses are closely related genetically to the NA genes of A/Moscow/10/99-like H3N2 viruses. Of the three substitutions observed in the NA genes of H1N2 viruses compared to those of A/Moscow/10/99, residues 199 and 431 are located in antigenic sites on surface of the NA gene (*33*,*34*). Residue 199 has also been assigned to one of the 12 phylogenetically important regions of the NA gene (*35*). All of the amino acid residues that form the sialic acid–binding site of the NA gene were conserved in the NA of H1N2 viruses. Although changes in receptor binding and the interaction between the HA and NA proteins of H1N2 reassortants may have facilitated the emergence of the H1N2 viruses, the contribution of the internal proteins to the replicative efficiency and transmission of these viruses and their interaction with the surface glycoproteins is unknown.

The early detection and characterization of newly emerging influenza variants are two of the primary aims of the World Health Organization global influenza surveillance network. Reassortment between circulating influenza viruses leading to the emergence of a novel subtype, such as H1N2, highlights the need for subtyping of the influenza A viruses isolated. Most current diagnostic tests rely on the detection and typing of the HA of influenza viruses. Only a few laboratories perform analysis of the NA gene (as NA inhibition assays are difficult to perform) or analyze the internal proteins and the genes encoding them. The use of molecular techniques provides a rapid means for the detection and subtyping of influenza viruses, although current PCR assays target only N1 and N2 subtypes (*22*,*36*). In addition, heteroduplex mobility assays can be used to genetically characterize the HA and internal genes of influenza viruses (*20*,*37*,*38*). However, although molecular methods aid the rapid detection and identification of influenza viruses, virus isolation by culture is still required for antigenic characterization of influenza viruses. How influenza H1N2 reassortants will evolve and whether H1N2 viruses will be maintained in circulation in humans remain to be seen.
